# Study on adsorption of kaolinite and gold thiosulfate

**DOI:** 10.1038/s41598-024-66389-z

**Published:** 2024-07-05

**Authors:** Zhonghang Chen, Pengcheng Li, Qianqian Wang, Shujuan Dai

**Affiliations:** School of Mining Engineering, University of Science and Technology Liaoning, Anshan, 114051 China

**Keywords:** Kaolinite, Gold-thiosulfate, Adsorption, Density functional theory, Solid Earth sciences, Mineralogy

## Abstract

The adsorption behavior of gold thiosulfate ions on the surface of kaolinite was studied using a combination of experimental research and quantum chemical calculations. Under the condition of a stirring time of 30 min, a stirring speed of 500 r·min^−1^, and a mass ratio of 30% kaolinite in the slurry, when the initial gold concentration of 56.50 mg·L^−1^,the adsorption rate of gold-thiosulfate ions from a kaolinite-containing solution was 7.44%. The results of Fourier transform infrared spectroscopy (FTIR) and energy dispersive spectroscopy (EDS) showed that the physical and chemical adsorption of kaolinite and gold thiosulfate occurred in solution. Quantum chemical calculations were performed using the CASTEP module in Materials Studio. The adsorption energy of gold thiosulfate on the surface of kaolinite (001) was calculated as − 438.01 kJ·mol^−1^.The calculated H76–O289 distance was 1.615 Å. Mulliken Charge population analysis and bond population analysis showed that gold thiosulfate ions form relatively stable bonds on the kaolinite surface (001). In the process of thiosulfate immersion, part of gold is adsorbed by kaolinite, which affects the extraction of gold. These results indicate that during the leaching process of gold thiosulfate, kaolinite has the ability to "catch" gold, which affects the leaching efficiency.

## Introduction

At present, although cyanide gold extraction is the most important, commonly used, and effective treatment process, which has received high attention from engineering and technicians, there are still serious problems with cyanide gold extraction. In the process of cyanide extraction, serious environmental pollution will occur, which greatly limits the application of this method^1–4^. With the increasing awareness of environmental protection, many scholars have proposed cyanide free leaching methods^5–7^. Thiosulfate leaching of gold is a simple, non-toxic, effective, and fast leaching method. Therefore, it is recommended as an alternative to traditional cyanide leaching technology^8^. Many scholars have conducted extensive research on the related field of thiosulfate leaching of gold. The research results indicate that thiosulfate forms a stable thiosulfate gold complex with gold.

The interaction between silicon mineral and gold has been studied by domestic and foreign scholars. During the research process, it was found that a large amount of gold was adsorbed or dissolved in siliceous minerals. Gold has relatively stable properties, but its interaction with silicon is very active. Silicon containing minerals produce organic silicon components through mechanical or chemical reactions during the beneficiation process. The interaction between the active silicone components and gold forms a strong chemical bond, which requires high energy to break it^9^.

There are many gangue minerals in the gold mine, mainly quartz and silicate minerals. Scientists have conducted numerous experiments on the interaction between these gangue minerals and gold. For example, Mohammadnejad et al.^10,11^ used different grinding methods to study the behavior of quartz prepregs and changed the pH value of the chloride medium to investigate how it affects the adsorption of gold by silicates. Diao Shuqin^12^ found that the gold content of clay minerals mainly hydromica in Qianxinan gold deposit was 93.71%, indicating that colloidal gold may be adsorbed by silicate minerals and clay minerals. Chen Yu et al.^13^ compared the adsorption rate changes of gold in solution with different particle sizes of muscovite through adsorption experiments, and explored the influence of muscovite on cyanide leaching rate through electrochemical dissolution experiments.

Kaolinite is the most common gangue mineral in gold mines, and the grinding process causes the interaction between gold, kaolinite, and silicate minerals to produce crystals. Scholars have conducted in-depth research on the interaction between gold and silicon, but there are few reports on the adsorption of kaolinite and gold under different stirring times. Our research team has conducted some experiments in this area.Senol Z M et al.^14^ studied the removal of uranyl ions from aqueous solution by kaolinite-based composite adsorbent,and made further dynamics and equilibrium analysis of this process. Enol Z M et al.^15^ explored the use of epichlorohydrin and tripolyphosphate-crosslinked chitosan-kaolin composites to remove auramine O dyes from aqueous solutions, on this basis, experimental research and DFT calculation are carried out. In the future, kaolinite can also be used in analytical technology and wastewater treatment technology and its application in sustainable environment.

Density functional theory (DFT) is a quantum mechanical method for studying multi electron systems. It is widely used in the fields of physics and chemistry, greatly simplifying quantum chemical calculations. DFT is used to calculate the surface properties of mineral crystals, analyze the microscopic state of minerals through surface calculations, and explain the macroscopic phenomena that occur during mineral processing. Using DFT to study the adsorption of gold thiosulfate ions on the surface of kaolinite can better explain experimental phenomena with basic theories^16–18^.

Hong Hanlie et al.^19^ studied the bonding between [Au(Sb_2_S_4_)]^−^ and the surface of kaolinite crystals through quantum mechanical simulations, and the results showed that [Au(Sb_2_S_4_)]^−^ forms covalent bonds with oxygen atoms in kaolinite crystals. Liu Shujie et al.^20^ explored the interaction between gold and kaolinite under mechanical activation, and conducted grinding experiments by thoroughly mixing gold chloride solution with kaolinite. AuCl_4_^−^ complex and kaolinite adsorption model was established, and quantum chemical simulation calculation was carried out to obtain the influence of mechanical activation on the interaction between gold and kaolinite. The gold complex interacted with silicate, which caused the loss of gold in the solution during hydrometallurgy; however, DFT has rarely been used to study the adsorption of gold thiosulfate ions on a kaolinite surface. Therefore, on the basis of experiments, using DFT to study the adsorption of gold thiosulfate ions on the surface of kaolinite can help us to understand this process more deeply. However, the gold thiosulfate ion is unstable during the experiment, so the testing time is strictly required.

This article adopts a combination of experimental research and quantum chemical calculations to study the ability of kaolinite to "catch gold" from non cyanide (thiosulfate) systems. Thus, the recovery rate of gold in the leaching process is affected. The experimental results provide a theoretical analysis for verifying the gold grabbing ability of kaolinite.

## Experimental

### Ore properties

**Figure 1:**
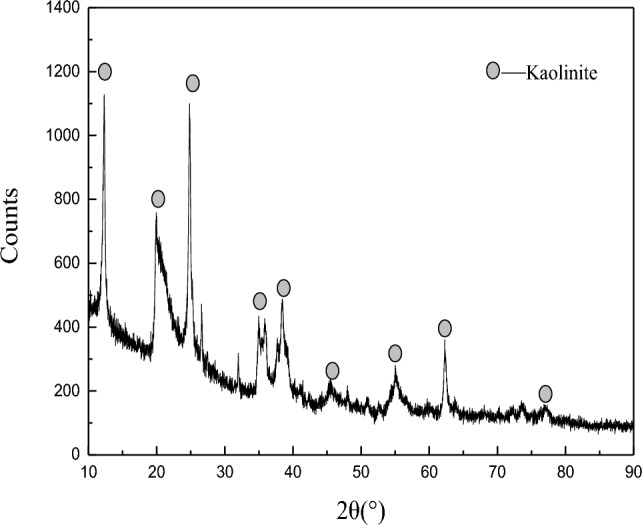
The XRD analysis results of the kaolinite.

The chemical composition of kaolinite samples was investigated by XRD (2θ range: 10°–90°), and the results are shown in Fig. 1 and Table 1.

**Table 1:** The multi-element analysis results of kaolinite.

Composition	Na_2_O	MgO	Al_2_O_3_	SiO_2_	P_2_O_5_	SO_3_	ZrO_2_	Cl
Content (%)	0.088	0.21	35	45.29	0.067	0.04	0.024	0.021
Composition	K_2_O	CaO	TiO_2_	Cr_2_O_3_	MnO	Fe_2_O_3_	WO_3_	NiO
Content (%)	0.094	0.298	1.37	0.012	0.012	2.205	0.01	0.006

As can be seen from Fig. 1 and Table 1, the main mineral is kaolinite. The main elements in the kaolinite sample are Si, Al and Fe, and the secondary elements are Ca, Mg and Ti, etc. The mass fraction of SiO_2_ is 45.29% and the mass fraction of Al_2_O_3_ is 35%, which meets the requirements of the single mineral test.

### Experimental methods

This experiment mainly studied the effect of stirring time on the unit adsorption capacity and adsorption rate of gold in solution. The samples were centrifuged, the solution was used to determine the concentration of gold and silicon by atomic absorption, and the solid was washed and dried for surface analysis. The amount of gold adsorbed is determined by the change of the mass concentration of gold in the solution.

Atomic absorption was used to determine the mass concentration of gold in the supernatant, so as to calculate the unit adsorption capacity and adsorption rate of kaolinite.

The washed and dried solids were analyzed by Fourier transform infrared spectroscopy (FTIR) and scanning electron microscopy/energy dispersive X-ray spectroscopy (SEM/EDS).

The samples before and after the adsorption of kaolinite with Au(S_2_O_3_)_2_^3–^ were analyzed by NICOLET 380 FT-IR spectrometer. The scanning electron microscope is ZEISS-σ IGMA HD from Zeiss, Germany.

The amount of gold absorbed is determined by changing the amount of gold in the solution. In order to quantify kaolinite and silicate minerals, the amount of gold adsorbed by kaolinite was used to determine the surface activity of silicon-containing minerals. Thiosulfate gold leaching was performed under alkaline conditions. In this experiment, NaOH was used to control the pH between 8 and 10. The gold removal efficiency α and unit-adsorption amount γ were calculated using the following equations:

1 $$\alpha = \frac{{c_{0} - c_{1} }}{{c_{0} }} \times 100{\text{\% ,}}$$

2 $$\gamma = \frac{{(c_{0} - c_{1} )v}}{{1000{\text{m}}}}.$$

where α (%) is the gold removal efficiency, γ (mg·g^−1^) is the unit adsorption capacity of gold, c_1_ (mg·L^−1^) is the concentration of gold in the supernatant, c_0_ (mg·L^−1^) is the initial gold solution concentration, *v* (mL) is the volume of gold solution, *m* is the mass (g) of the kaolinite sample.

The convergence criteria of the ideal structure and for the energy calculation are as follows: (a) an energy tolerance of 2 × 10^−5^ eV/atom, (b) a maximum-force tolerance of 0.05 eV/Å, (c) a maximum-displacement tolerance of 0.002 Å and (d) a self-consistent-field tolerance of 1.0 × 10^−6^ eV/atom).

### Experimental results

**Figure 2:**
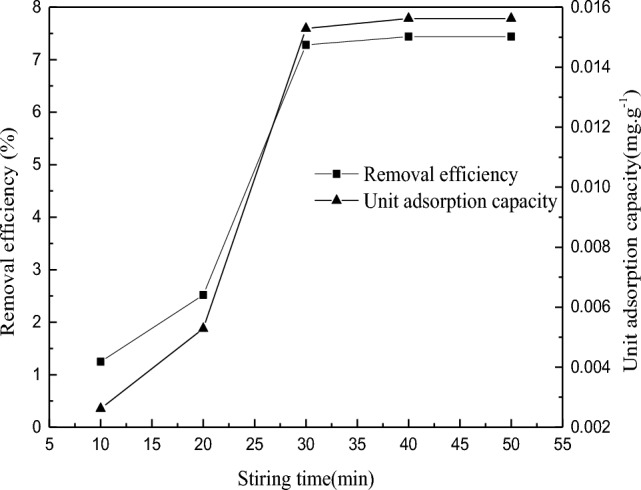
Effect of stirring time on adsorption of kaolinite.

The adsorption rate of kaolinite in gold thiosulfate solution was determined by examining the stirring time. The result is shown in Fig. 2.

Under the experimental conditions that the fineness of kaolinite is – 0.037 mm, the mass fraction is 100%, the ore content is 5 g, the pulp mass fraction is 30%, the stirring speed is 500r/min, and the initial gold solution mass concentration is 56.50 mg/L, the adsorption rule of kaolinite in the solution is studied by changing the stirring time. When the stirring time is between 10 and 30 min, the unit adsorption capacity and removal efficiency of kaolinite to gold in solution increase with the increase of stirring time, and the adsorption effect is enhanced. The removal efficiency reached 7.44% at about 30 min. After 30 min, with the increase of stirring time, the unit adsorption capacity and removal efficiency were basically unchanged, and the adsorption effect was weakened.

### The adsorption mechanism of gold thiosulfate ions on the surface of kaolinite

#### Change of FTIR spectra before and after the kaolinite adsorption

**Figure 3:**
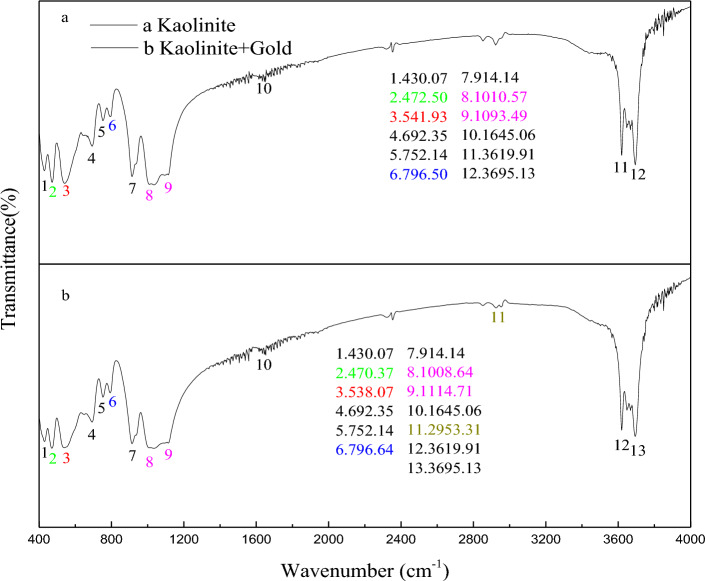
Infrared spectrum before and after kaolinite adsorption.

The FTIR spectra of kaolinite and gold thiosulfate are shown in Fig. 3.

From Fig. 3, it can be concluded that the Si–O–Al stretching vibration peak is at 541.93 cm^–1^, the Si–O bending vibration peak is at 472.50 cm^–1^ and 430.07 cm^–1^, and the Al–O stretching vibration and –OH translation at these two wave numbers also contribute. The peak of Si–O–Si symmetric stretching vibration in the weak band is 796.50 cm^–1^ and 752.14 cm^–1^, and the peak of Si–O–Si symmetric stretching vibration is 692.35 cm^–1^. The wobble absorption peak of outer hydroxyl group was 914.14 cm^–1^. 1093.49 cm^–1^and 1010.57 cm^–1^ are shoulder-shaped, strong absorption bands, which are Si–O stretching vibration peaks; 1645.06 cm^–1^ is the bending vibration absorption peak of water molecules. There are two sharp absorption bands in the high frequency region, and the stretching vibration absorption peaks of the outer and inner hydroxyl groups of kaolinite are at 3695.13 cm^–1^and 3619.91 cm^–1^, respectively.

By comparing Fig. 3a and b, it can be seen that some peaks changed after Au (S_2_O_3_)_2_^3–^ is adsorbed by kaolinite. After the reaction, the Si–O–Al stretching vibration peak at 541.93 cm^–1^ red shifted to 538.07 cm^–1^, and the Si–O bending vibration peak at 472.50 cm^–1^, while the Al–O stretching vibration peak and –OH translation also contributed, red shifted to 470.57 cm^–1^ after the reaction. It shows that hydrogen bond and weak intermolecular force are produced. After the reaction, the symmetric stretching vibration peak of Si–O–Si at 796.50 cm^–1^ was red shifted to 792.64 cm^–1^. The absorption peak at 1093.49 cm^–1^ was shoulder-shaped and was a strong absorption band Si–O stretching vibration peak. After the reaction, the bending vibration absorption peak of water molecules at 1645.06 cm^–1^ was blue shifted to 1114.71 cm^–1^, 1010.57 cm^–1^ was red shifted to 1008.64 cm^–1^. In the high frequency region 3695.13 cm^–1^, 3619.91 cm^–1^, the outer hydroxyl group and the inner hydroxyl group expansion vibration absorption peak did not change before and after reaction. The above results show that Au (S_2_O_3_) _2_^3–^ can be adsorbed by kaolinite, and it is physical adsorption process. As can be seen from Fig. 3b, a new peak is formed at 2953.31 cm^–1^ after the solution of kaolinite and Au (S_2_O_3_)_2_^3–^ is stirred, indicating that the chemical adsorption of kaolinite and Au (S_2_O_3_)_2_^3–^ occurs.

#### Change of EDS analysis before and after kaolinite adsorption

**Figure 4:**
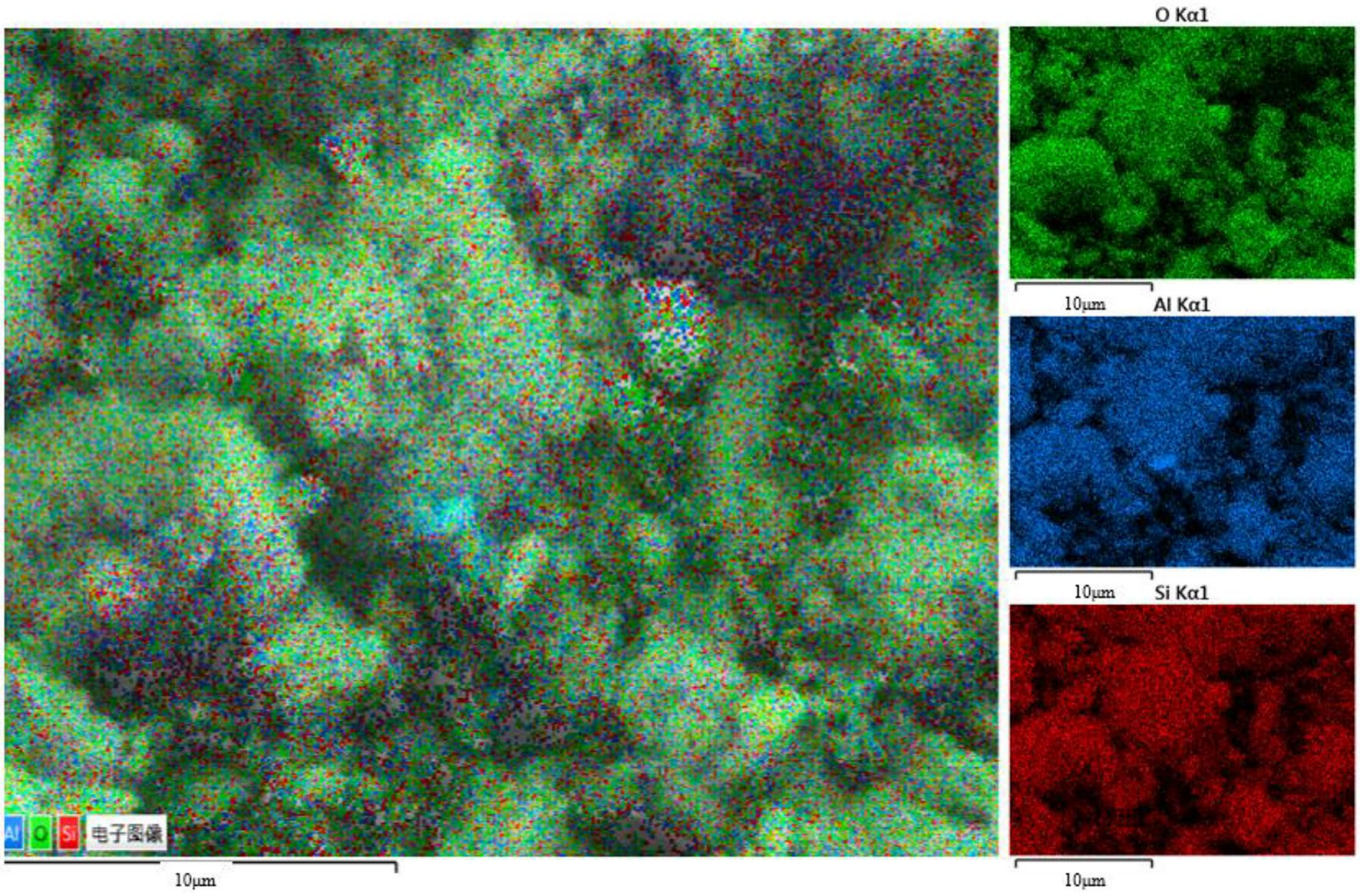
Hierarchical analysis of kaolinite scanning electron microscopy EDS.

**Figure 5:**
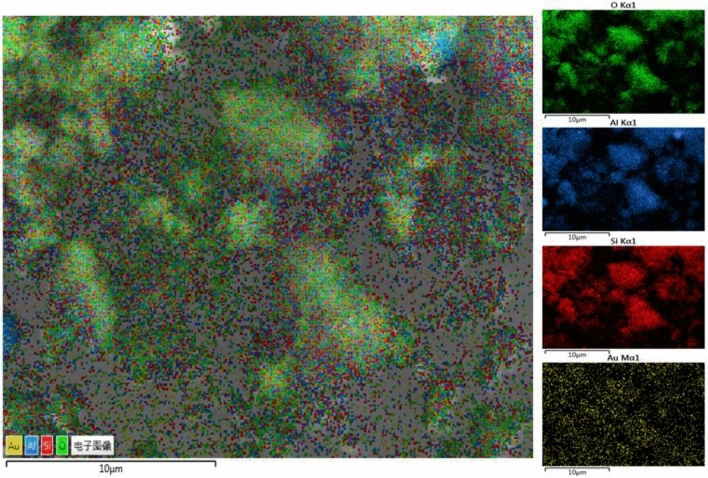
Stratified analysis of scanning electron microscopy EDS after kaolinite and Au(S_2_O_3_)_2_^3–^ adsorption.

The SEM/EDS analysis of kaolinite before and after adsorption is shown in Figs. 4 and 5.

In Figs. 4 and 5, The EDS layered image clearly and uniformly displays Si, Al, and O elements, indicating the presence of strong chemical bonds between Si, Al, and O in kaolinite. Some Au atoms are uniformly dispersed between Si and O atoms, indicating that during the adsorption reaction between kaolinite and gold thiosulfate solution, some gold atoms are adsorbed on the surface of kaolinite.

## Quantum calculations and analysis

The structure of kaolinite was optimized using the CASTEP module in Materials Studio, and the band, density of electronic states and Mulliken populations were calculated.The surface of kaolinite was relaxed to find a suitable cleavage surface. Then, the molecular surface interaction model is developed, and quantum mechanical calculations and analysis were conducted.

### Establishment of of the ideal crystal structure of kaolinite

#### The Kaolinite crystal convergence test

Based on the optimization of the original kaolinite cell model, the optimal exchange correlation function, K-point and plane wave truncation energy are determined. Then, the correlation function of GGA-PBESOL is selected, the k point is 3 × 2 × 2, and the truncation energy is 500 eV. Under this parameter, the total energy of the system is − 8656.44 eV, and the lattice length error is 0.75%. The optimized volume parameters and experimental results of kaolinite cells are shown in Table 2.

**Table 2:** Kaolinite experiment and calculation optimization comparison table.

Parameter	Experiment	Simulation	Difference (%)
A (Å)	5.149	5.184	0.68
B (Å)	8.934	9.001	0.75
C (Å)	7.384	7.330	0.74

**Figure 6:**
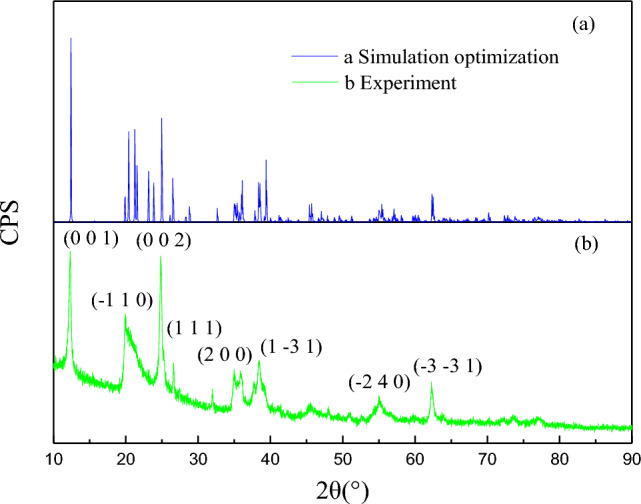
Comparison of simulated and experimental XRD spectra.

Reference^21^ shows that the optimized lattice parameter data are reasonable, and the simulation results show that the calculation method and the selected parameters are reliable. Due to the use of pseudo-potentials, the lattice parameters *a* are overestimated by about 0. 68%, *b* are overestimated by about 0.75%, while *c* is overestimated by 0. 74%. Figure 6a and Fig. 6b show the simulated and experimental XRD patterns respectively.

Figure 6 compares the simulated parameters with the experimental XRD patterns. The (001) and (002) planes of Kaolinite represent the formation of stable kaolinite crystals.

It has a dominant dissociation surface (Fig. 6b). The (001) plane has the lowest energy, so subsequent calculations use this plane^22^.

#### Band structure and density of states analysis of kaolinite crystals

**Figure 7:**
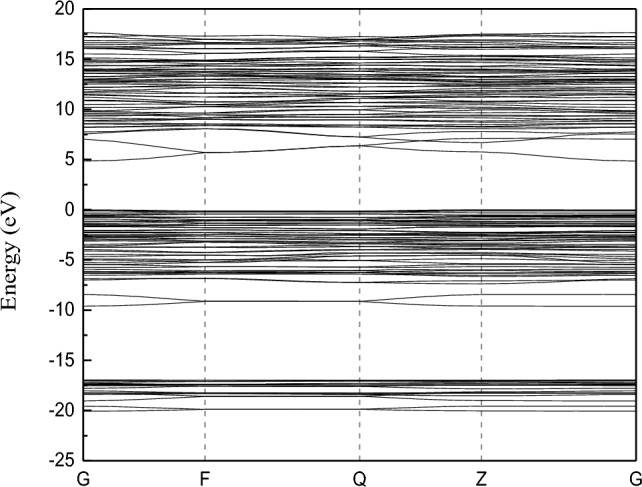
Band structure of kaolinite.

**Figure 8:**
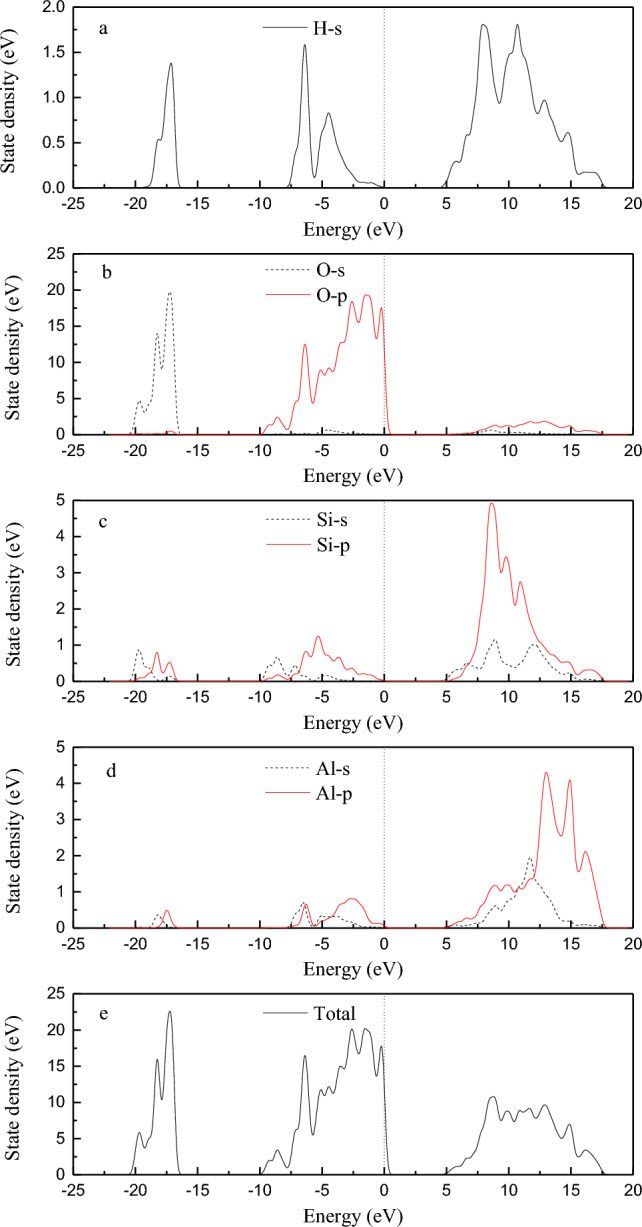
Density of states of kaolinite.

The structure of the kaolinite band and the density of states is shown by Figs. 7 and 8.

First, the Fermi level is is determined as the zero energy. Figure 7 shows that the forbidden band width of kaolinite is 4.850 eV. The bandwidth of semiconductors is generally below 2 eV, while the bandwidth of insulators is relatively large; Therefore, kaolinite is classified as an insulator.

From Fig. 8, it can be seen that the energy band of kaolinite is divided into three parts. The first one is the *s* orbits of hydrogen atoms and oxygen atoms which play a role in the valence band between − 20 and − 15 eV. The second one is the *s* orbit of hydrogen atom, the *p* orbital of oxygen atom and the *p* orbit of silicon atom which contribute the valence band between − 10 and 2.5 eV, and the *p* orbit of oxygen atom contributes the most. And the third one is the *s* orbits of hydrogen atom, *s* and *p* orbits of silicon atom and *s* and *p* orbits of aluminum atom which contribute between 5 and 17.5 eV, and the *p* orbits of silicon atom and aluminum atom contribute more. The conduction band energy level is mainly contributed by the *s* orbits of hydrogen atom, *s* orbits of silicon, *p* orbits and *s* orbits and *p* orbits of aluminum atom. The valence band at the Fermi level is mainly contributed by the *s* orbital of hydrogen atom and the *p* orbital of oxygen. Therefore, the interaction between kaolinite and Au(S_2_O_3_)_2_^3–^ ions during adsorption is mainly caused by the *s* orbit of hydrogen atom and the *p* orbit of oxygen atom.

#### Analysis of the Mulliken populations of the kaolinite crystals

The valence electron configurations of H, O, Si and Al atoms in kaolinite before optimization were hydrogen 1s^1^, oxygen 2s^2^2p^4^, silicon 3s^2^3p^2^, aluminum 3s^2^3p^1^, respectively. Table 3 shows the optimized atomic populations.

**Table 3:** Mulliken atomic population analysis of kaolinite.

Mineral	Atom	*s*	*p*	Total (e)	Charge (e)
Kaolinite	H	0.57	0	0.57	0.43
	O	1.85	5.26	7.11	− 1.11
	Si	0.62	1.17	1.79	2.21
	Al	0.47	0.73	1.20	1.80

It can be seen from Table 3 that the optimized electron configuration of kaolinite is hydrogen 1s^0.57^, oxygen 2s^1.85^2p^5.26^, silicon 3s^0.62^3p^1.17^ and aluminum 3s^0.47^3p^0.73^. Hydrogen atom, silicon atom and aluminum atom are electron donors, hydrogen *s* orbit, silicon *s* orbital and *p* orbital, aluminum *s* orbital and *p* orbital lose electrons, localized in hydrogen atom electron number is 0.57e, lose 0.43e, hydrogen atom charge number is + 0.43e. The number of electrons of silicon atom is 1.79e, losing 2.21e, and the number of electric charge of silicon atom is + 2.21e. The aluminum atom has a number of electrons of 1.20e and loses 1.80e, and the silicon atom has a charge of + 1.80e. Oxygen is an electron acceptor, and oxygen loses electrons in *s* orbits, but the number of electrons gained by *p* orbits is much higher than the number of electrons lost by *s* orbits. The oxygen atom has a charge of − 1.11e. The *p* orbits of oxygen atom is the most active orbits.

### Calculation of the interaction between the (001) surface of kaolinite and gold-thiosulfate ions

#### Calculation of optimal surface layer of kaolinite (001) surface

Surface energy is defined as the reversible work per unit surface area, and the lower the value indicates the more stable surface and accurate surface structure. The thickness of the atomic layer is 5.292– 40.692 Å, and the thickness of the vacuum layer is 10–20 Å. The formula for calculating surface energy is given in reference^23^.

The initial thickness of the vacuum layer is set to 20 Å, and the surface energy of kaolinite (001) with different atomic layer thicknesses is calculated. Table 4 shows the results.

**Table 4:** Surface energy of different atomic layer thicknesses.

Atomic-layer thickness (Å)	5.292	12.372	19.452	26.532	33.612	40.692
Surface energy (J·m^–2^)	0.164	0.221	0.210	0.192	0.174	0.159

From Table 4, it can be seen that when the atomic layer thickness reaches 12.372 Å, the surface energy change of kaolinite is less than 0.05 J·m^–2^, and it has reached a steady state. Therefore, for subsequent calculations, the atomic layer thickness was set to 26.532 Å. According to the thickness of the atomic layer, the surface energy of the kaolinite (001) surface is calculated. Table 5 shows the results.

**Table 5:** Surface energy of different vacuum layer thicknesses.

Vacuum layer thickness (Å)	10	12	14	16	18	20
Surface energy (J·m^–2^)	0.174	0.181	0.185	0.188	0.190	0.192

**Figure 9:**
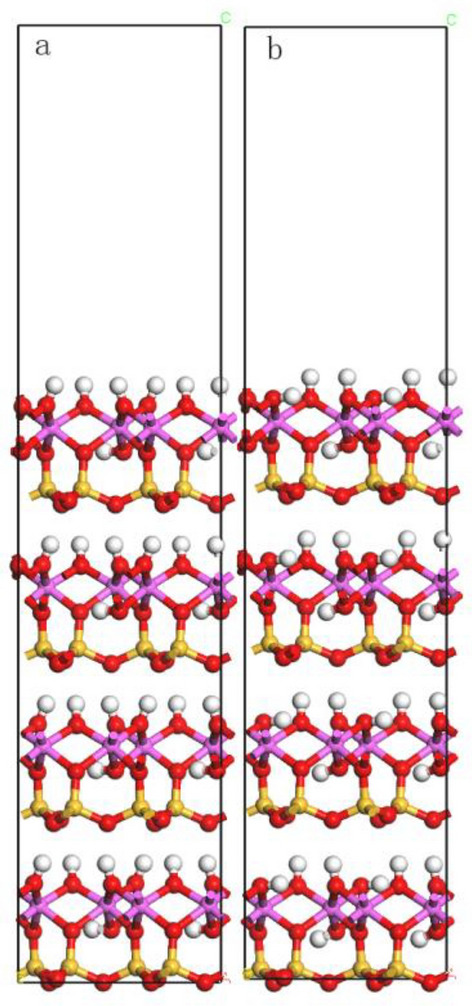
The unit cell model of the kaolinite (001) surface before relaxation (**a**) and after relaxation (**b**).

According to Table 5, changing the thickness of the vacuum layer has little effect on surface energy, and the surface of kaolinite reaches a stable state. For overall considerations, the thickness of the vacuum layer was set to 16 Å. Figure 9 shows the surface model before and after relaxation, and the model after relaxation was used for subsequent adsorption simulations.

#### Adsorption of the adsorbed material on the surface of kaolinite (001)

The adsorption energy on the surface of kaolinite (001) was calculated using CASTEP simulation. A negative adsorption energy indicates exothermic adsorption and that the adsorbate had a substantial effect on the kaolinite surface. The formula for calculating the total adsorption energy is given in reference^24^.

Sulfosulfate leaching of gold is usually carried out under ammonia conditions, so hydroxide ions and water molecules can affect the adsorption process of Au (S_2_O_3_)_2_^3–^ ions on the surface of kaolinite (001) in solution. This simulation calculates the energy of the interaction between water molecules, hydroxide ions, Au (S_2_O_3_)_2_^3–^ ions and kaolinite (001) surface. Prior to adsorption, water molecules, hydroxide ions, Au (S_2_O_3_)_2_^3–^ ions are placed in 10 × 10 × 10 Å cubic cells for optimal optimization. In this case, The gamma point was selected as the *k*-point.

**Figure 10:**
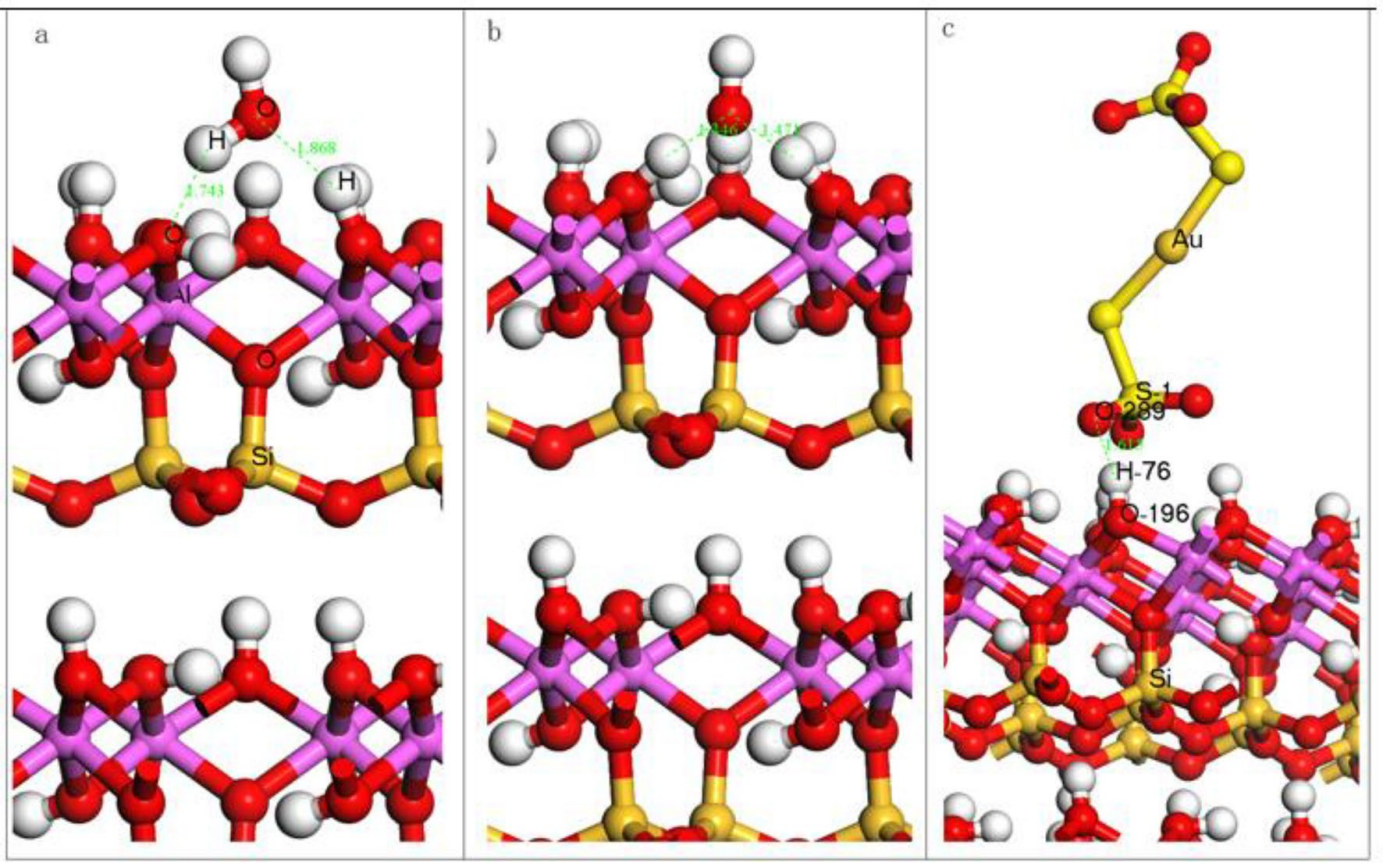
Geometric adsorption structure of water molecule (**a**) hydroxide ion (**b**) Au(S_2_O_3_)_2_^3-^ ion (**c**) on kaolinite.

The geometric adsorption structures of hydroxyl ions, water molecules and Au (S_2_O_3_)_2_^3–^ ions optimized on the surface of kaolinite are shown in Fig. 10, and the calculated adsorption energies are shown in Table 6.

**Table 6:** Adsorption energy of adsorbate on kaolinite (001) surface.

Adsorbate	H_2_O	OH^-^	Au(S_2_O_3_)_2_^3-^
Adsorption energy (kJ·mol^–1^)	− 33.51	− 480.91	− 438.01

According to Fig. 10 and Table 6, the adsorption energy of Au (S_2_O_3_)_2_^3–^ ions on the surface of kaolinite (001) is − 438.01 kJ·mol^−1^, and the O–H distance is 1.615 Å, indicating that Au(S_2_O_3_)_2_^3–^ ions can naturally adsorb on the surface of kaolinite (001). The adsorption energy of water molecules is − 33.51 kJ·mol^−1^, which indicates that water molecules can be adsorbed on the surface of kaolinite (001) and form a hydration film on the surface of kaolinite (001). The adsorption energy of OH^−^ ions on the surface of kaolinite (001) is − 480.91 kJ·mol^−1^, with strong interaction and the formation of hydrogen bonds. In summary, the adsorption capacity from strong to weak is OH– > Au (S_2_O_3_)_2_^3–^ > H_2_O.

#### Electronic structure analysis of kaolinite crystals

Gold-thiosulfate ions adsorbed on the (001) surface of kaolinite. Table 7 shows that adsorption occurred between the H-76 and O-289 atoms.

**Table 7:** Au(S_2_O_3_)_2_^3–^ Mulliken population analysis on kaolinite (001) surface.

原子		S orbit	P orbit	Total (e)	Charge (e)
S-1	Before adsorption	1.21	2.63	3.84	2.16
	After adsorption	1.25	2.66	3.92	2.08
O-289	Before adsorption	1.89	5.12	7.00	− 1.00
	After adsorption	1.89	5.04	6.93	− 0.93
H-76	Before adsorption	0.52	–	0.52	0.48
	After adsorption	0.58	–	0.58	0.42
O-196	Before adsorption	1.84	5.23	7.06	− 1.06
	After adsorption	1.84	5.21	7.04	− 1.04

It can be seen from Table 7, after the adsorption of Au (S_2_O_3_)_2_^3–^ ions on the surface of kaolinite (001), the 3 s and 3 *p* orbital electrons of S-1 atom increase from 1.21 e to 1.25 e and 2.63 e to 2.66 e, respectively, and the total electrons increase from 3.84 to 3.92 e, an increase of 0.08e. The 2 s orbit electrons of O-289 atoms do not change, while the 2* p* orbit decreases from 5.12 to 5.04 e, and the total electrons decrease from 7.00 to 6.93 e, a decrease of 0.07 e. The 1 s orbit of H-76 atom increases from 0.52 to 0.58 e, and the total electron increases from 0.52 to 0.58 e, an increase of 0.06 e. The O-196 atom has no change in the 2 s orbit electrons, while the 2 *p* orbit decreases from 5.23 to 5.21 e, and the total electron decreases from 7.06 to 7.04 e, a decrease of 0.02 e. This shows that the electron part of the O-289 atom on the Au (S_2_O_3_)_2_^3–^ ion is converted to the H-76 atom on the kaolinite surface. The electron part of the O-289 atom in the Au (S_2_O_3_)_2_^3–^ ion is converted to the S-1 atom of the Au (S_2_O_3_)_2_^3–^ ion, The electron portion of the O-196 atom on the surface of kaolinite is transferred to H-76. The population analysis of bonds adsorbed by Au (S_2_O_3_)_2_^3–^ ions on the surface of kaolinite (001) is shown in Table 8.

**Table 8:** Analysis of the bond of Au(S_2_O_3_)_2_^3–^ ion adsorbed on the surface of kaolinite (001).

Bond		Population	Bond length (Å)
S1-O289	Before adsorption	0.51	1.460
	After adsorption	0.50	1.487
H76-O289	Before adsorption	0.13	1.615
H76-O196	After adsorption	0.56	0.976
	Before adsorption	0.53	1.010

The binding strength can be expressed by the relative number of overlapping Mulliken populations, and the more Mulliken overlapping populations, the stronger the covalent property of the bond. When the overlapping population is small, the electron cloud overlap is small, and if the number of charged atoms gradually increases, the bond exhibits ionic character.

As can be seen from Fig. 10c and Table 8: After the reaction of kaolinite with Au (S_2_O_3_)_2_^3–^ ion, the length of S1-O289 bond in Au (S_2_O_3_)_2_^3–^ ion increases from 1.460 to 1.487 Å, and the length of H76-O196 bond on kaolinite (001) surface increases from 0.976 to 1.010 Å. This shows that the bond length of O289 and H76 becomes shorter during the interaction, and a reaction occurs, that is, O289 in Au (S_2_O_3_)_2_^3–^ ion forms a bond with H76 on the surface of kaolinite (001), the population value is 0.13, and the bond length is 1.615 Å. From the population value and bond length, the O atom in Au (S_2_O_3_)_2_^3–^ ion is bonded to the H atom on the surface of kaolinite (001), and the bonding effect is relatively weak.

## Conclusions

1. After kaolinite adsorbed Au(S_2_O_3_)_2_^3–^ solution, kaolinite and Au(S_2_O_3_)_2_^3–^ occurred physical adsorption and chemical adsorption, and part of Au atoms adsorbed on the surface of kaolinite.
2. According to the energy band, state density and Mulliken population analysis of kaolinite crystals, the valence band of Fermi level is mainly contributed by the s orbital of hydrogen atom and the p orbital of oxygen.
3. Mulliken charge and bond population analysis showed the electron portion of the O-289 atom on the Au(S_2_O_3_)_2_^3–^ ion is converted to the H-76 atom on the kaolinite surface, and the O-289 in the Au(S_2_O_3_)_2_^3–^ ion is bonded to the H-76 on the kaolinite (001) surface with a population value of 0.13 and a bond length of 1.615 Å which were consistent with the experimental results.
4. During gold adsorption, some gold was adsorbed by silicate minerals such as kaolinite, which affected the gold extraction and recovery rates. By studying the ability of kaolinite to "catch" gold during the process of thiosulfate leaching of gold, theoretical analysis is provided for kaolinite as a catching material.
5. In the future research work, we will study the highly efficient leaching reagent, establish the mechanism model of leaching reagent and gold or silicon, optimize the leaching conditions, reduce the influence of silicate minerals such as kaolinite on the leaching effect of gold, and thus improve the recovery rate of gold.

## Data Availability

All data generated or analysed during this study are included in this published article.
